# Beyond knowledge gains - learner experience and technology acceptance of virtual reality for anatomy education in Zambia: a randomized exploratory study

**DOI:** 10.1186/s41077-026-00493-0

**Published:** 2026-10-31

**Authors:** Sandra Barteit, Nizar El Ahmadie, Jessica Nieder, Petros Andreadis

**Affiliations:** 1 Heidelberg Institute of Global Health (HIGH), Faculty of Medicine and University Hospital, Heidelberg University, Im Neuenheimer Feld 130.3, 69120 Heidelberg, Germany; 2 Leeds and York Partnership NHS Foundation Trust, Leeds, UK

**Keywords:** Virtual reality, Anatomy education, Technology Acceptance Model (TAM), User experience, Low-resource setting

## Abstract

**Background:**

Standalone virtual reality (VR) enables interactive exploration of three-dimensional anatomy, but comparative evidence from resource-constrained medical-education settings remains limited. This study explored immediate knowledge outcomes, learner acceptance, and experiences with VR and three other learning resources in Zambia.

**Methods:**

In this four-arm randomized exploratory study, 67 first- and second-year Bachelor of Science in Clinical Sciences students were assigned using Excel-generated random group numbers (1–4; no allocation concealment) to standalone VR using 3D Organon, AMBOSS^®^, curated textbook materials, or self-directed internet search for a self-paced learning session of up to 30 min. Knowledge was assessed using an unvalidated 10-item pre-post test, and immediate acceptance was measured with an adapted Technology Acceptance Model questionnaire. The primary between-group knowledge analysis used baseline-adjusted analysis of covariance with HC3-robust standard errors. Acceptance comparisons were adjusted for multiple testing. Brief open-text responses underwent qualitative content analysis. A pre-study review indicated that tested concepts could be located in the three fixed-content resources but did not establish equivalence in depth, organisation, or accessibility, and could not be applied to the self-directed internet group.

**Results:**

The knowledge analysis included 59 participants (VR *n* = 12; AMBOSS^®^ *n* = 17; textbook *n* = 16; Internet *n* = 14). Within-group increases reached the adjusted significance threshold in the AMBOSS^®^, textbook, and web-resource groups but not in the VR group (adjusted *p* = .053). The primary analysis did not detect an overall difference in baseline-adjusted post-test scores between modalities, F(3,54) = 1.945, *p* = .133; none of the six pairwise comparisons was significant after Holm adjustment. This finding does not establish equivalence. Acceptance analyses included 55–60 participants per construct. After adjustment across six domains, only Attitude differed significantly between modalities, H(3) = 11.801, ε² = 0.17, adjusted *p* = .049; AMBOSS^®^ was rated more favourably than the self-directed internet search (adjusted *p* = .019, Cliff’s δ = −0.60). Perceived ease of use and perceived enjoyment showed low internal consistency, limiting their interpretation. The qualitative corpus comprised 211 comments from 64 participants. Some VR participants valued visualisation, novelty, and enjoyment but reported navigation and content-access difficulties. AMBOSS^®^ participants frequently valued organisation and accessibility, while experiences with textbook and web resources were mixed.

**Conclusions:**

This small exploratory study did not provide conclusive evidence of differences in immediate knowledge outcomes between the four modalities. Quantitative acceptance differences were also limited after adjustment for multiple comparisons. The findings identify content accessibility, curricular alignment, interface familiarization, and exposure time as priorities for larger, prospectively designed studies using validated instruments and longer-term outcomes.

**Supplementary Information:**

The online version contains supplementary material available at https://doi.org/10.1186/s41077-026-00493-0.

## Introduction

Virtual reality (VR) is expanding the pedagogical toolkit of medical education by enabling immersive and interactive exploration of three-dimensional structures [1, 2]. In anatomy education, learners using VR can rotate models, alter viewing perspectives, isolate structures, and examine spatial relationships that may be less readily explored using static textbook illustrations or web images. Such functionality may complement established approaches, particularly where access to hands-on anatomy resources is limited [3, 4]. Standalone head-mounted displays may reduce some logistical barriers associated with earlier VR systems because they do not require tethering to a high-performance computer [5–7], and development work has increasingly emphasized cost-effective, scalable platforms [8]. Nevertheless, their implementation still requires investment in hard- and software, reliable charging, maintenance and hygiene arrangements, technical support, learner orientation, and content aligned with the local curriculum [5, 7].

Evidence concerning the educational effects of VR is promising. In a systematic review and meta-analysis across health-professions education, VR was associated with a small improvement in post-intervention knowledge compared with traditional learning (standardized mean difference [SMD] 0.44, 95% confidence interval [CI] 0.18–0.69; moderate-certainty evidence) and a larger improvement in cognitive skills (SMD 1.12, 95% CI 0.81–1.43; moderate-certainty evidence) [2]. These estimates were derived from heterogeneous interventions, learner populations, and outcomes, and their applicability to anatomy education specifically is therefore uncertain. Other reviews have similarly reported variable findings across applications and study designs [1, 9, 10].

The potential relevance of scalable educational resources must also be considered within the broader health-workforce context. Health-professions education in sub-Saharan Africa is constrained by shortages of qualified teaching faculty, limited teaching and learning materials, and educational infrastructure restrictions [3, 4]. The World Health Organization (WHO) projects a global shortfall of approximately 11 million health workers by 2030, concentrated predominantly in low- and middle-income countries (LMICs) [11], a projection revised from the 18 million estimated in the 2016 global strategy on human resources for health [12]. Within the WHO African Region, an estimated 3 million additional health workers would need to be educated and trained by 2030 beyond the number expected under current trajectories [4]. Zambia illustrates these workforce constraints: WHO reported 2.95 medical doctors per 10 000 population in 2021, compared with 45.03 in Germany in the same year and a global average of 17.2 in 2022 [13]. At the same time, regional initiatives have demonstrated the potential of digitally mediated education to extend access to health-worker training: a collaboration between the WHO Regional Office for Africa and Project ECHO delivered 287 virtual learning sessions between April 2020 and February 2024, recording more than 125,000 attendances, compared with fewer than 2,000 participants reached annually through face-to-face training before the pandemic. Continued investment in digital infrastructure, technology, and learner support nevertheless remains necessary [14].

Evidence about immersive technologies in resource-constrained settings remains limited. A systematic review of head-mounted extended-reality applications in medical education found that 25 of 27 included studies were conducted in high-income countries, two in middle-income countries, and none in low-income countries [9]. A subsequent critical review identified a growing but still heterogeneous body of VR and augmented-reality research in LMICs, with limited evidence to guide sustainable implementation [5]. Early work in Kenya found substantial learner enthusiasm but also identified technical barriers associated with systems that required powerful computers and external tracking equipment [6]. Comparative evidence concerning newer standalone headsets in low-resource anatomy education therefore remains scarce [5, 6, 9].

Adoption cannot be inferred from educational performance alone. Perceived usefulness, ease of use, attitudes, and intention to continue using a resource may influence whether an educational technology is incorporated into routine learning. The Technology Acceptance Model provides a framework for examining these factors [15, 16], and a recent systematic review found growing use of this framework in medical education, although its application to immersive technologies remains comparatively limited [17]. In resource-constrained settings, acceptance may also depend on practical usability, access, technical reliability, and the alignment of available content with local curricula and assessments [5, 7].

The present study was designed as an exploratory, hypothesis-generating four-arm randomized comparison incorporating quantitative outcomes and an embedded analysis of brief written open-text comments. It aimed to: (1) describe short-term pre–post knowledge-score changes within groups and explore between-group differences in post-test scores adjusted for baseline across standalone VR, AMBOSS^®^, curated textbook materials, and self-directed internet search; (2) describe learner-reported technology acceptance and explore differences across the four modalities; and (3) examine participants’ written comments concerning their learning experiences, usability, content alignment, and implementation-related challenges. Given the modest group sizes and exploratory design, the study was not intended to establish equivalence, comparative effectiveness, or policy suitability. Rather, it provides preliminary estimates and contextually grounded observations to inform the design of subsequent, adequately powered studies using explicitly aligned educational content.

## Methods

### Reporting guidelines

**Fig. 1:**
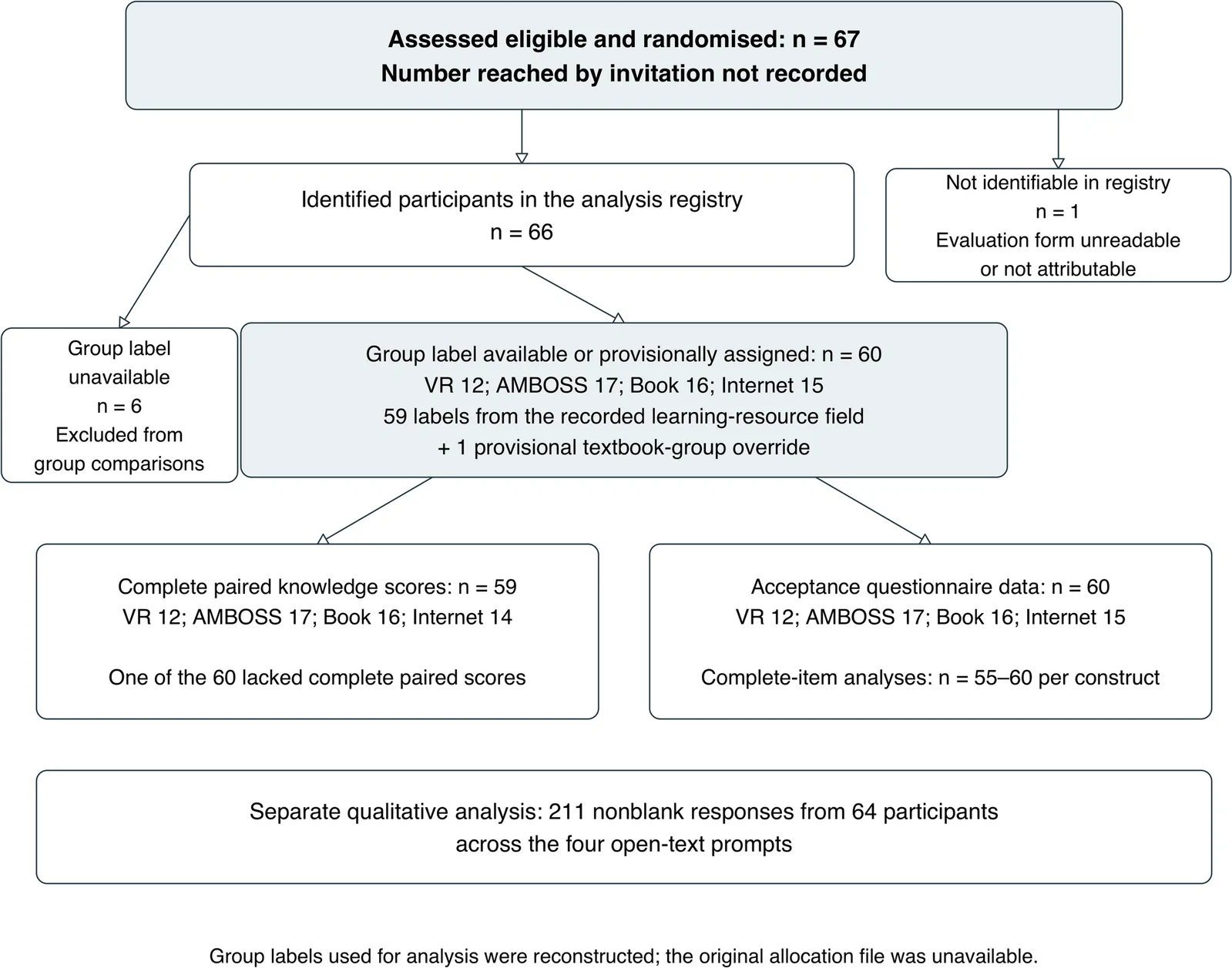
Participant flow and analysis populations. Of 67 randomised participants, 66 could be identified in the analysis registry; one evaluation form could not be read or attributed. Group labels used for analysis were reconstructed from recorded learning resources and one provisional textbook-group override; the original allocation file was unavailable. Knowledge n = 59; acceptance *n* = 60 (55–60 per construct); qualitative analysis: 211 responses from 64 participants. VR, virtual reality

This report was prepared with reference to the CONSORT 2025 statement for randomized trials [18]; the completed checklist is provided in Appendix C, and participant flow is shown in Fig. 1. CONSORT was used as a reporting framework rather than as a characterization of the study as a clinical treatment trial. Because the qualitative material comprised brief written responses to open-ended survey questions rather than interviews or focus groups, the Standards for Reporting Qualitative Research (SRQR) were used as the principal qualitative reporting framework [19]. A COREQ checklist [20] is additionally provided in Appendix D for completeness.

### Study design

This was an individually randomized, four-arm, parallel-group exploratory educational study examining short-term anatomy knowledge and learner acceptance following use of standalone VR, AMBOSS, textbook materials or self-directed internet search. The study used a quantitatively dominant design with a supplementary qualitative component comprising brief written responses to open-ended survey questions.

The four modalities were selected to represent two resource formats commonly used by students at Levy Mwanawasa Medical University (LMMU), respectively textbook and web-based materials, and two distinct technology-enhanced approaches: curated e-learning and immersive three-dimensional visualization with HMDs. Including all four arms allowed preliminary estimation of several potentially relevant contrasts within the same student cohort but reduced the number of participants available per arm. The design was therefore intended to identify patterns and comparisons for subsequent research, not to establish equivalence, non-inferiority or comparative effectiveness.

### Participants and study setting

The study was conducted in July 2022 at Levy Mwanawasa Medical University (LMMU) in Lusaka, Zambia. Eligible participants were first- and second-year students enrolled in the Bachelor of Science in Clinical Sciences program who, according to the curriculum schedule, had not yet received formal instruction in gastrointestinal anatomy. Individual prior informal study of gastrointestinal anatomy was not assessed.

Students were recruited through a WhatsApp invitation distributed by the LMMU student secretary’s office. The number of students who received the invitation was not recorded. Sixty-seven students volunteered, were assessed as meeting the eligibility criteria, provided written informed consent before undertaking any study procedures, and were randomized. Participation was voluntary and did not affect students’ grades, course progression, or access to educational resources. No financial or academic incentive was provided.

### Randomization, allocation concealment, and blinding

After enrolment had closed, the participant list was organised by gender and sorted by age. Microsoft Excel was then used to generate a random group number from 1 to 4 for each participant, with the four numbers corresponding to VR, AMBOSS^®^, textbook, or self-directed internet search. No blocked, sequential, repeating, or formally stratified allocation procedure was used.

The assignments were generated by the study lead (SB), who therefore knew the intervention assignments before the study sessions. No independent allocation service, sequentially numbered sealed envelopes, or other formal allocation-concealment mechanism was used. Participants and proctors were informed of the intervention assignment only after all participants in a study round had completed the pre-test. The original Excel allocation spreadsheet was not retained and could therefore not be independently reviewed during manuscript revision.

Because the learning resources were visibly different, participants and proctors could not be blinded after assignments were disclosed. Knowledge tests carried participant codes only and were scored against a prespecified answer key by an assessor blinded to group assignment.

### Interventions

All participants received the same study guide specifying six learning objectives concerning the stomach: location, topographical relationships, anatomical parts, blood supply, innervation, and lymphatic drainage. The objectives were displayed in both study rooms throughout the learning session.

Each intervention was self-paced and limited to a maximum of 30 min. Participants were told they could end the session earlier once they considered their study of the specified objectives complete. Time-on-task was not systematically recorded; the study therefore compared the resources under self-paced conditions rather than under conditions of equal exposure time.

**Fig. 2:**
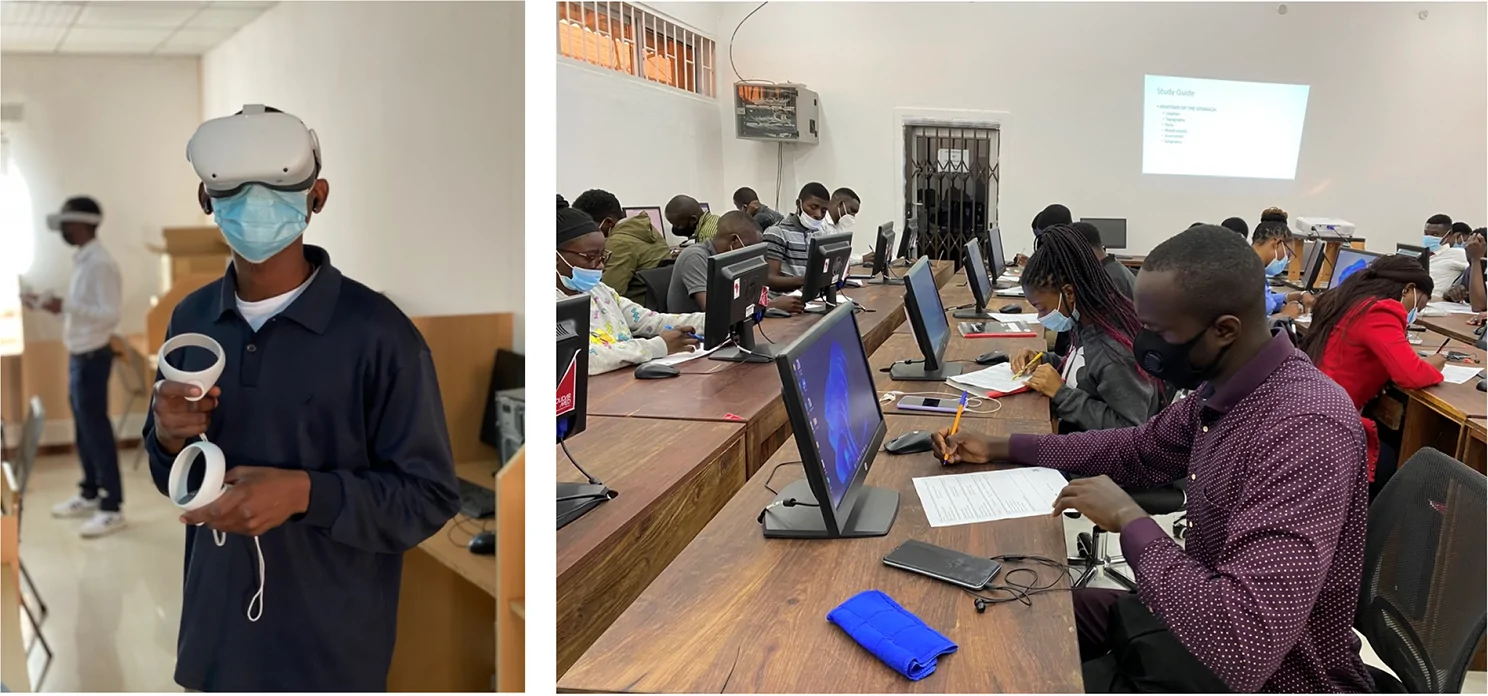
Study settings and learning setup. Left: a participant using the 3D Organon Anatomy 2022 Edition application on a Meta Quest 2 headset in the separate VR room; printed operating instructions were available, and the room provided space for movement during headset use. Right: the main computer laboratory, with the study guide and learning objectives projected at the front of the room

All sessions were proctored at LMMU. Proctors ensured that participants used only their allocated resource and provided procedural and technical assistance only, not anatomy instruction. Participants assigned to AMBOSS^®^, the textbook, or self-directed internet search worked in the main computer laboratory under the supervision of four proctors. Participants assigned to VR used a separate room, supervised by one proctor, that provided sufficient space for safe movement during headset use. The study settings and displayed learning objectives are illustrated in Fig. 2.

1. **Virtual reality**: Participants used the 3D Organon Anatomy 2022 Edition application on one of three Meta Quest 2 standalone headsets (Meta Platforms, USA). Because a maximum of three participants could use the headsets concurrently, VR participants completed the intervention in sequential subgroups of up to three. Before beginning, participants received a 5-minute standardized orientation covering headset adjustment, controller use, navigation, and safety instructions; the orientation addressed operation of the equipment and did not include teaching about stomach anatomy. Using the hand controllers, participants could rotate the anatomical model, alter the viewing perspective, and isolate or hide individual structures.
2. **AMBOSS®**: Participants accessed the AMBOSS® e-learning platform through institutional licenses in Mozilla Firefox version 102 on university desktop computers with 21-inch monitors. Direct links were provided to the AMBOSS® library articles and anatomical illustrations addressing the six learning objectives.
3. **Textbook**: Participants received printed extracts from Gray's Anatomy for Students, 4th edition, selected by an LMMU anatomy lecturer to address the six learning objectives.
4. **Internet**: Participants used the same hardware and browser as the AMBOSS® group and were free to search the internet for material addressing the six learning objectives. No specific websites were prescribed. Because participants selected their own sources, the content accessed in this group was not standardised and was not recorded.

The AMBOSS^®^ and self-directed internet search interventions required the university’s internet connection, whereas the VR and textbook interventions could be used offline. The sequence of assessments within each study round is described under Study Procedure.

### Pre-study content review

Before the study, two LMMU medical teachers informally reviewed the VR application, AMBOSS, and textbook extracts with reference to the six curricular learning objectives and the knowledge-test content. No structured item-by-resource mapping or formal audit record was created or retained. The Internet condition could not be reviewed because participants selected their own online sources and the content accessed was neither standardized nor recorded. This informal review therefore did not establish equivalence in content depth, terminology, organization, visibility, or accessibility for novice learners.

### Data collection instruments

#### Knowledge test

Anatomy knowledge was assessed with the same 10-item multiple-choice test (Appendix A) before the intervention and, after all participants in a study round had completed their learning session, at post-test (see Study Procedure). Each correct response received one point; incorrect responses, and items verified against the original paper forms as unanswered, received zero, producing a total score of 0–10. Tests with no scorable responses were treated as missing rather than scored zero.

The research team developed the items against the six curricular learning objectives. Two LMMU medical lecturers reviewed them for curricular relevance, clarity, and accuracy of the answer key. The same test was used at both time points to avoid difficulty differences between parallel forms. No answers or item-level feedback were given after the pre-test, but repeated exposure to the same questions may have produced recall or testing effects.

#### Acceptance questionnaire

Learner acceptance was assessed after the intervention using a paper-based questionnaire (Appendix B), adapted principally from the Technology Acceptance Model 3 (TAM3) [15]; because Attitude is not retained as a central construct in TAM3, the Attitude items were adapted from the original Technology Acceptance Model [16]. Appendix B identifies the source construct and wording adaptation for each item.

Responses were recorded on a five-point Likert-type scale from 1 (“strongly agree”) to 5 (“strongly disagree”); after reverse scoring the negatively worded Perceived Ease of Use item Q7, lower scores indicated more favourable perceptions. The analysis covered Perceived Usefulness, Perceived Ease of Use, Attitude, Behavioural Intention, Job Relevance, and Perceived Enjoyment. Job Relevance comprised a single item and was analysed as such rather than as a reliability-tested scale. Q21 and Q23 were excluded from the primary Attitude and Behavioural Intention scores because they duplicated Q13 and substantially overlapped Q16, respectively. These exclusions were made post hoc during peer-review revision; sensitivity analyses retaining the items are described under Data Analysis. Three further items were analysed separately and assigned to no TAM domain: Q4 (perceived adequacy of study time), Q5 (perceived alignment between materials and test), and Q22 (self-perceived knowledge improvement). No overall acceptance score was calculated, as the questionnaire comprised conceptually distinct domains and was not validated as a unidimensional scale.

### Study procedure

Because of capacity limitations in the computer laboratory and VR room, the study was conducted in three consecutive rounds on the same day. The study lead scheduled participants across the three rounds and provided the student secretary and class representative with the attendance times to be communicated to participants. They received only the time-slot information and were not informed of the participants’ intervention assignments. Participants were therefore notified of their attendance time in advance, but their assigned learning resource was not disclosed until after completion of the pre-test.

Students had volunteered and been enrolled before the study day. Each round followed the same sequence: on arrival, participants received information about the study and provided written informed consent before any data were collected, then completed the 10-item knowledge pre-test. The pre-generated intervention assignments were then disclosed, and participants moved to the room designated for their resource.

Participants completed a self-paced learning session of no more than 30 min. Those who finished early returned their assigned resource and waited in a supervised area without access to further learning materials and were asked not to discuss the session while waiting. Once all participants in the round had finished, everyone completed the post-test followed by the acceptance questionnaire under proctored conditions. To reduce contamination between rounds, participants in earlier rounds were asked not to discuss the learning materials, study procedures, or knowledge-test content with participants scheduled for later rounds. Adherence was not formally assessed.

### Data analysis

Data management and quantitative analyses were performed in R version 4.3.3 using a documented, scripted procedure; no raw data files were modified, and all corrections were applied in analysis code. Correct responses received one point; incorrect responses, verified unanswered items, and items with more than one mark received zero (score range 0–10). No unresolved items remained after form review, and no values were imputed.

The primary between-group analysis was an analysis of covariance with post-test score as the outcome, baseline score as covariate, and intervention group as a categorical predictor, using HC3 heteroskedasticity-consistent standard errors given the small, unequal groups. The overall group effect was tested with a robust omnibus F-test; baseline-adjusted means and all six pairwise differences were estimated with 95% confidence intervals and Holm-adjusted p-values. To examine whether adjustment for gender materially changed the estimates, an exploratory gender-adjusted complete-case model was run as a sensitivity analysis. Because gender was missing for one analysed participant, the full-sample baseline-adjusted model remained the primary analysis. Change scores were also compared between groups by two-sided Kruskal-Wallis test with epsilon-squared (ε²); Dunn pairwise comparisons with Holm adjustment were specified only if that omnibus test was significant. Within-group pre–post changes were tested with two-sided Wilcoxon signed-rank tests, Holm-adjusted across the four groups, reporting Hodges-Lehmann estimates and matched-pairs rank-biserial correlations with 95% confidence intervals. These within-group results are not evidence of intervention efficacy, as the study had no no-intervention control.

Because the knowledge test had not been psychometrically evaluated, exploratory item analyses (item facility, corrected item–total correlations, Kuder–Richardson 20) were conducted separately for the pre- and post-test and interpreted cautiously given the small sample, ten-item length, and repeated use of the same instrument.

Acceptance items were scored 1 (strongly agree) to 5 (strongly disagree), with lower scores more positive; Q7 was reverse-coded. Construct scores were the mean of Perceived Ease of Use (Q6, reverse-coded Q7), Perceived Usefulness (Q8-Q11), Attitude (Q12-Q13), Behavioural Intention (Q14-Q16), Job Relevance (Q17), and Perceived Enjoyment (Q18-Q20), calculated only when all constituent items were available; participants could contribute to one construct despite missing another. Q21 and Q23, identified during revision as duplicating Q13 and Q16, were excluded from the primary definitions, with analyses retaining them reported as sensitivity analyses. No overall acceptance score was calculated, as the questionnaire comprised conceptually distinct domains and was not validated as unidimensional.

Internal consistency was estimated with the Spearman-Brown coefficient for two-item constructs and Cronbach’s alpha for constructs of three or more items, with 95% bootstrap confidence intervals (2,000 resamples); no coefficient was computed for single-item Job Relevance. Construct scores were compared across groups by Kruskal-Wallis test with ε² reported for every comparison, Holm-adjusted across the six omnibus tests. For constructs significant after adjustment, Dunn pairwise comparisons were Holm-adjusted within the six comparisons of that construct, with Cliff’s delta and bootstrap 95% confidence intervals as pairwise effect sizes. For analysis, 59 intervention-group labels were taken from the recorded learning-resource field, with one additional provisional assignment to the textbook group. These reconstructed labels were not verified against the original randomisation list, which was unavailable. The six acceptance omnibus tests were repeated after excluding the single provisional override, and Holm adjustment was reapplied to that six-test family.

The qualitative corpus comprised 211 nonblank responses from 64 respondents across four prompts (53 pre-test reflections, 52 post-test reflections, 58 comments on resource helpfulness, 48 further comments) and was analysed by one researcher (JN, a health-psychology researcher at the Heidelberg Institute of Global Health) using qualitative content analysis. Responses were first classified deductively by evaluative direction (positive, negative, or for test-related responses mixed); the reasons underlying these evaluations were then coded inductively and grouped into higher-order categories, examined across the whole corpus and within each intervention group, with test-related responses compared before and after the intervention. The resulting framework organised resource-related responses around affective experience and perceived usefulness, the latter comprising system quality or ease of use and information quality (organisation and visualisation of information; relevance and completeness of content). Records were compiled in Microsoft Word and the framework summarised in XMind. All tests were two-sided, with significance defined as *p* < .05 after the stated multiplicity adjustment. Given the exploratory design and modest group sizes, emphasis was placed on effect estimates, confidence intervals, and consistency across analyses; non-significant findings are not interpreted as evidence of equivalence.

## Results

### Descriptive study results

Of the 67 randomised participants, 66 could be identified in the analysis registry; one evaluation form could not be read or attributed. Group labels were available or provisionally assigned in the analysis files for 60 of these participants, while six had no usable group label. Of these, 59 also had complete pre- and post-intervention knowledge scores and were included in the primary knowledge analysis, and all 60 had questionnaire data and were included in the acceptance analyses; the number contributing to each construct ranged from 55 to 60 because complete responses to all constituent items were required. The qualitative corpus comprised 211 nonblank responses contributed by 64 participants across the four open-text prompts.

Participants were first- and second-year Bachelor of Science in Clinical Sciences students at LMMU. In the primary knowledge-analysis sample, 49 of 59 (83.1%) were aged 20–24 years and 33 (55.9%) were male; age data were missing for two participants and gender for one. Participant characteristics are summarized in Table 1, and participant flow and analysis populations are presented in Fig. 1.

**Table 1:** Participant characteristics by intervention group

Characteristic	VR (*n* = 12)	AMBOSS^®^ (*n* = 17)	Book (*n* = 16)	Internet (*n* = 14)	Total (*N* = 59)
*Age group, n (%)*					
20–24 years	11 (91.7)	12 (70.6)	16 (100.0)	10 (71.4)	49 (83.1)
≥ 25 years	1 (8.3)	3 (17.6)	0 (0.0)	4 (28.6)	8 (13.6)
Missing	0 (0.0)	2 (11.8)	0 (0.0)	0 (0.0)	2 (3.4)
*Gender*,* n (%)*					
Female	5 (41.7)	5 (29.4)	6 (37.5)	9 (64.3)	25 (42.4)
Male	7 (58.3)	11 (64.7)	10 (62.5)	5 (35.7)	33 (55.9)
Missing	0 (0.0)	1 (5.9)	0 (0.0)	0 (0.0)	1 (1.7)
*Pre-intervention knowledge score (0–10)*					
Mean (SD)	2.67 (1.97)	3.41 (1.73)	4.06 (1.06)	3.64 (1.65)	3.49 (1.64)
Median (IQR)	2.5 (1.0–4.0)	4.0 (2.0–5.0)	4.0 (3.0–5.0)	3.5 (2.3–4.8)	4.0 (2.0–5.0)

Data are n (%) unless otherwise stated. The table describes the knowledge-analysis population (N = 59); the acceptance analyses included 60 participants

Percentages are calculated column-wise, using the total number of participants in each column as the denominator, and therefore include participants with missing demographic data. Percentages may not sum to exactly 100% because of rounding

IQR, interquartile range; SD, standard deviation; VR, virtual reality

### Quantitative results

#### Knowledge scores

**Fig. 3:**
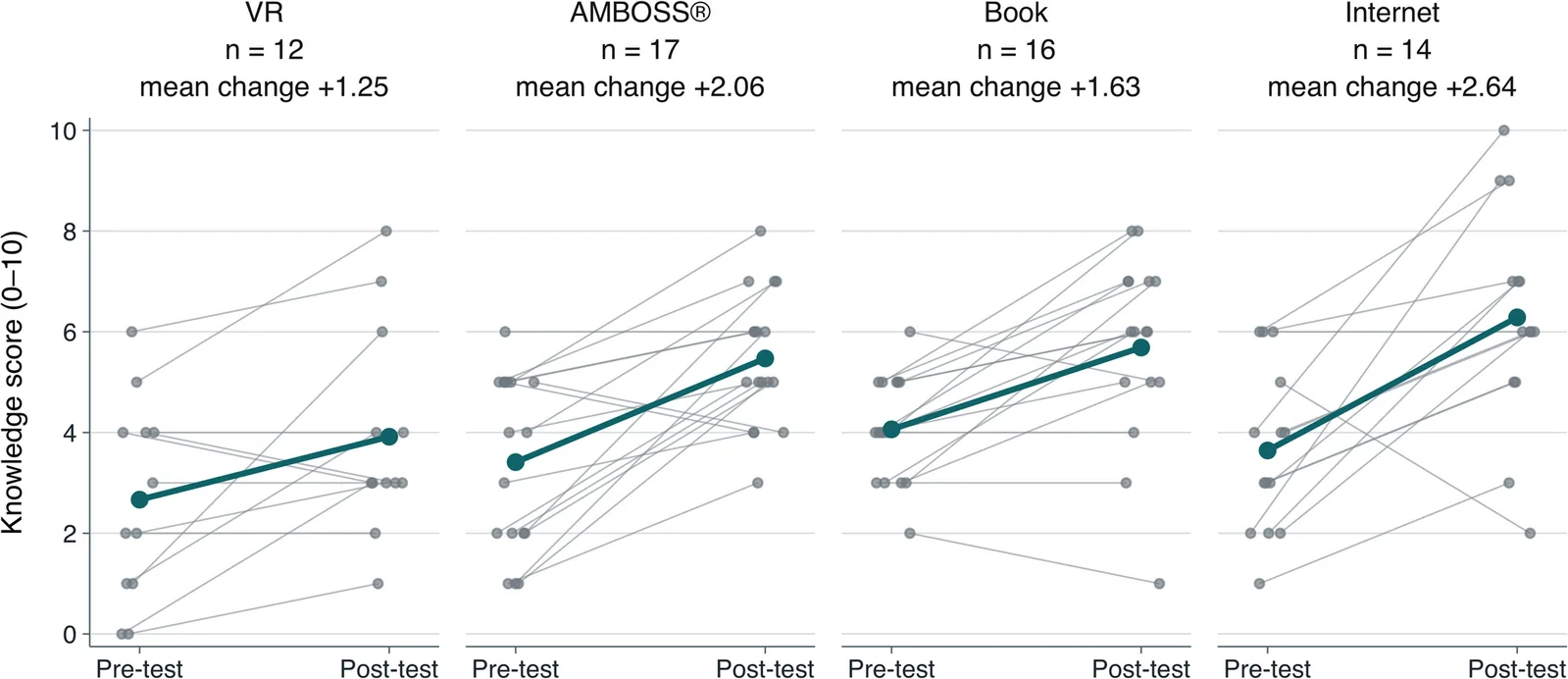
Individual pre- and post-intervention knowledge scores (0–10) by learning modality. Grey lines show individual participants, offset slightly to separate coincident integer scores; teal lines show unadjusted group means. Group sizes are shown in each panel

Median knowledge scores increased descriptively from pre-test to post-test in all four groups: from 2.5 (IQR 1.0–4.0) to 3.0 (IQR 3.0-4.5) in the VR group, from 4.0 (IQR 2.0–5.0) to 5.0 (IQR 5.0–6.0) in the AMBOSS^®^ group, from 4.0 (IQR 3.0–5.0) to 6.0 (IQR 5.0–7.0) in the Book group, and from 3.5 (IQR 2.25–4.75) to 6.0 (IQR 5.25-7.0) in the Internet group (Table 2). Individual pre- and post-test trajectories are shown in Fig. 3.

After Holm adjustment across the four within-group Wilcoxon signed-rank tests, evidence of a pre-post increase was observed in the AMBOSS^®^ group (Hodges-Lehmann estimated change: 2.0 points, 95% CI 1.0–3.0; adjusted *p* = .006), Book group (2.0 points, 95% CI 1.0–3.0; adjusted *p* = .011), and Internet group (3.0 points, 95% CI 1.5–4.5; adjusted *p* = .017). The corresponding result for the VR group did not meet the 0.05 threshold after adjustment (2.0 points, 95% CI 0.0–3.0; adjusted *p* = .053).

The primary between-group analysis used an analysis of covariance with post-test score as the outcome and baseline score as a covariate. The HC3-robust omnibus test did not provide statistically significant evidence of an overall intervention-group effect, F(3, 54) = 1.945, *p* = .133. Baseline-adjusted post-test means were 4.26 (95% CI 3.12–5.41) for VR, 5.50 (95% CI 4.84–6.17) for AMBOSS^®^, 5.45 (95% CI 4.51–6.38) for Book, and 6.22 (95% CI 4.98–7.46) for Internet (Table 2). None of the six pairwise comparisons was statistically significant after Holm adjustment (all adjusted *p* ≥ .144).

A Kruskal-Wallis analysis of change scores, conducted as a sensitivity analysis, produced a consistent conclusion, H(3) = 3.799, *p* = .284, ε² = 0.015. The study therefore did not detect a statistically significant difference in short-term knowledge outcomes between the four modalities. Given the exploratory design, small group sizes, and wide confidence intervals, this result does not demonstrate equivalence or rule out potentially meaningful differences between modalities. The complete-case gender-adjusted HC3 sensitivity model included 58 participants and likewise did not detect an overall group effect, F(3,52) = 2.336, *p* = .0845. Exploratory internal consistency of the 10-item knowledge test was KR-20 = .32 at pre-test and .54 at post-test (*n* = 59 at each time point).

#### Acceptance ratings

**Fig. 4:**
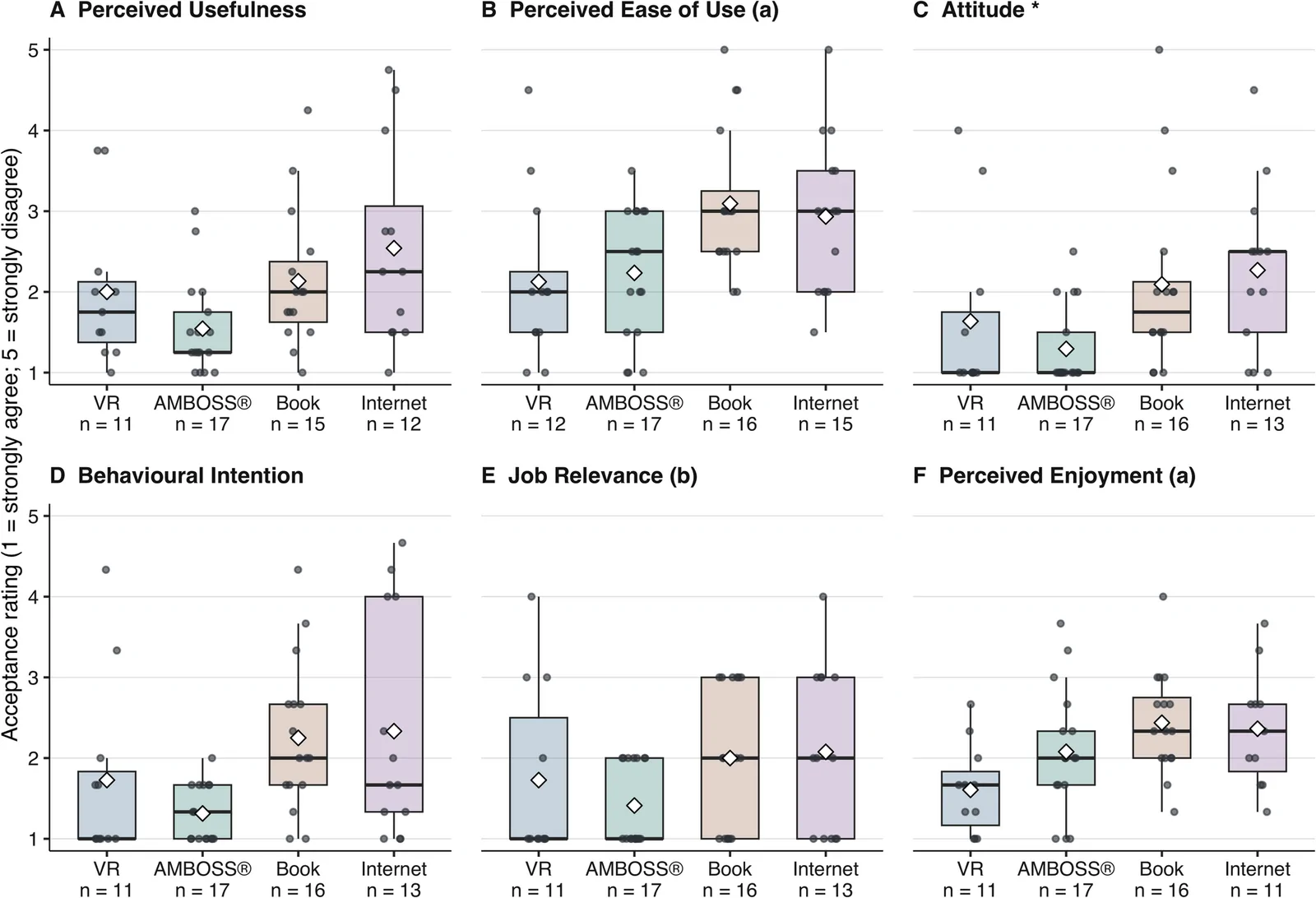
Acceptance ratings by construct and learning modality. Boxes show medians and interquartile ranges (IQR); whiskers extend to the most extreme observations within 1.5 × IQR of the box. Diamonds show group means and points show individual participants (jittered horizontally). Lower ratings are more favourable (1 = strongly agree, 5 = strongly disagree). Sample sizes are shown beneath each box. *Significant after Holm adjustment across the six omnibus tests. (a) Low internal consistency; interpret cautiously. (b) Single-item measure; internal consistency was not estimated

Acceptance analyses included between 55 and 60 participants, depending on item-level missingness. Scores ranged from 1 (strongly agree) to 5 (strongly disagree), lower scores represented more favourable ratings. The distribution of ratings by domain is shown in Fig. 4, and full results are reported in Table 3.

Before correction for multiple testing, omnibus Kruskal-Wallis p-values were below 0.05 for five of the six assessed domains. After Holm adjustment across the six omnibus tests, however, only Attitude retained statistical significance, H(3) = 11.801, ε² = 0.166, adjusted *p* = .0486; ε² values for all six domains are reported in Table 3. In the subsequent Dunn comparisons, the AMBOSS^®^ group reported a more favourable attitude than the Internet group: the median attitude score was 1.0 (IQR 1.0-1.5) for AMBOSS^®^ and 2.5 (IQR 1.5–2.5) for Internet (Holm-adjusted *p* = .019; Cliff’s δ = −0.60, 95% CI − 0.88 to − 0.27). No other pairwise attitude comparison reached statistical significance after adjustment (all adjusted *p* ≥ .056). The remaining omnibus comparisons did not meet the adjusted significance threshold, although Behavioural Intention fell only marginally short: Behavioural Intention (adjusted *p* = .0503), Perceived Ease of Use (adjusted *p* = .0607), Perceived Enjoyment (adjusted *p* = .0607), Perceived Usefulness (adjusted *p* = .0626), and the single-item Job Relevance measure (adjusted *p* = .191). In an exploratory sensitivity analysis excluding the participant assigned to the textbook group through a provisional override, the Attitude analysis included 56 participants, H(3) = 11.411, Holm-adjusted p = .0582 across the six construct tests. No construct met the .05 threshold in this sensitivity analysis. When Q21 and Q23 were retained in the Attitude and Behavioural Intention scores, respectively, both analyses included 57 participants. Attitude remained significant, H(3) = 13.659, Holm-adjusted p = .0205, whereas Behavioural Intention did not, H(3) = 9.213, Holm-adjusted p = .0798. Holm adjustment was reapplied across all six constructs, with the other four construct definitions unchanged. Internal consistency estimates (95% bootstrap confidence intervals; construct-complete n) were: Perceived Usefulness, Cronbach’s alpha .87 [.75, .92], *n* = 55; Perceived Ease of Use, Spearman–Brown .31 [−.28, .63], n = 60; Attitude, Spearman–Brown .90 [.79, .96], *n* = 57; Behavioural Intention, Cronbach’s alpha .92 [.85, .95], *n* = 57; and Perceived Enjoyment, Cronbach’s alpha .39 [.01, .61], *n* = 55. Intervals used 2,000 bootstrap resamples. Job Relevance was a single-item measure, so internal consistency was not estimated (*n* = 57).

Overall, the acceptance findings suggest a possible difference in attitudes, particularly a more favourable attitude toward AMBOSS^®^ than toward self-directed internet search, but do not establish a general preference for either technology-enhanced modality.

**Table 2:** Knowledge outcomes by intervention group

Outcome	VR (*n* = 12)	AMBOSS^®^ (*n* = 17)	Book (*n* = 16)	Internet (*n* = 14)
*Pre-test score*				
Mean (SD)	2.67 (1.97)	3.41 (1.73)	4.06 (1.06)	3.64 (1.65)
Median [IQR]	2.50 [1.00–4.00]	4.00 [2.00–5.00]	4.00 [3.00–5.00]	3.50 [2.25–4.75]
*Post-test score*				
Mean (SD)	3.92 (2.07)	5.47 (1.33)	5.69 (1.85)	6.29 (2.20)
Median [IQR]	3.00 [3.00–4.50]	5.00 [5.00–6.00]	6.00 [5.00–7.00]	6.00 [5.25–7.00]
*Change score (post − pre)*				
Mean (SD)	1.25 (1.86)	2.06 (1.82)	1.63 (1.59)	2.64 (2.53)
Median [IQR]	1.00 [0.00–3.00]	2.00 [1.00–3.00]	2.00 [0.75–3.00]	2.00 [2.00–4.00]
*Within-group pre–post comparison*				
Hodges–Lehmann change [95% CI]	2.00 [0.00–3.00]	2.00 [1.00–3.00]	2.00 [1.00–3.00]	3.00 [1.50–4.50]
Rank-biserial correlation [95% CI]	0.73 [0.29–0.92]	0.90 [0.72–0.96]	0.89 [0.69–0.96]	0.84 [0.54–0.95]
Wilcoxon p, Holm-adjusted	0.053	0.006	0.011	0.017
*Between-group comparison*				
Baseline-adjusted post-test mean [95% CI]	4.26 [3.12–5.41]	5.50 [4.84–6.17]	5.45 [4.51–6.38]	6.22 [4.98–7.46]

CI, confidence interval; IQR, interquartile range; SD, standard deviation; VR, virtual reality. Confidence intervals are pointwise 95% intervals without multiplicity adjustment. Wilcoxon p-values are Holm-adjusted across the four within-group comparisons

**Table 3:** Acceptance ratings and omnibus comparisons by intervention group

Construct	VR	AMBOSS^®^	Book	Internet	H(3)	ε²	Holm-adj. *p*
Perceived Usefulness	*n* = 11 2.00 (0.94) 1.75 [1.38–2.13]	*n* = 17 1.54 (0.59) 1.25 [1.25–1.75]	*n* = 15 2.13 (0.87) 2.00 [1.63–2.38]	*n* = 12 2.54 (1.26) 2.25 [1.50–3.06]	8.853	0.115	0.063
Perceived Ease of Useᵃ	*n* = 12 2.13 (1.05) 2.00 [1.50–2.25]	*n* = 17 2.24 (0.81) 2.50 [1.50–3.00]	*n* = 16 3.09 (0.92) 3.00 [2.50–3.25]	*n* = 15 2.93 (0.96) 3.00 [2.00–3.50]	10.441	0.133	0.061
**Attitude**	*n* = 11 1.64 (1.10) 1.00 [1.00–1.75]	*n* = 17 1.29 (0.50) 1.00 [1.00–1.50]	*n* = 16 2.09 (1.14) 1.75 [1.50–2.13]	*n* = 13 2.27 (1.03) 2.50 [1.50–2.50]	**11.801**	**0.166**	**0.0486**
Behavioural Intention	*n* = 11 1.73 (1.12) 1.00 [1.00–1.83]	*n* = 17 1.31 (0.34) 1.33 [1.00–1.67]	*n* = 16 2.25 (0.94) 2.00 [1.67–2.67]	*n* = 13 2.33 (1.39) 1.67 [1.33–4.00]	11.330	0.157	0.0503
Job Relevanceᵇ	*n* = 11 1.73 (1.10) 1.00 [1.00–2.50]	*n* = 17 1.41 (0.51) 1.00 [1.00–2.00]	*n* = 16 2.00 (0.89) 2.00 [1.00–3.00]	*n* = 13 2.08 (1.04) 2.00 [1.00–3.00]	4.753	0.033	0.191
Perceived Enjoymentᵃ	*n* = 11 1.61 (0.55) 1.67 [1.17–1.83]	*n* = 17 2.08 (0.77) 2.00 [1.67–2.33]	*n* = 16 2.44 (0.64) 2.33 [2.00–2.75]	*n* = 11 2.36 (0.72) 2.33 [1.83–2.67]	10.436	0.146	0.061

Within each intervention-group cell, results are presented as n, mean (SD), and median [IQR]. Scores ranged from 1 (strongly agree) to 5 (strongly disagree); lower values therefore indicate more favourable perceptions. Construct-specific sample sizes vary because complete responses to all constituent items were required

^a^ Low internal consistency; interpret cautiously

^b^ Single-item measure; internal consistency was not estimated

IQR, interquartile range; SD, standard deviation; VR, virtual reality

### Qualitative analysis

The qualitative corpus comprised 211 nonblank responses contributed by 64 participants across four open-text prompts: 53 pre-test reflections, 52 post-test reflections, 58 comments concerning the helpfulness of the assigned learning resource, and 48 additional comments about the learning experience. Responses were analysed using deductive–inductive qualitative content analysis. Comments were first classified as positive, negative, or mixed, and participants’ reasons for these evaluations were then coded inductively and organized into broader categories. The coding framework organised resource-related responses around three principal categories: affective experience and engagement; system quality and ease of use; and information quality, including the organisation, visualisation, relevance, and completeness of the content. Perceived usefulness cut across these categories. Comments concerning the knowledge test were considered separately in relation to perceived difficulty, clarity and fairness, preparation, and perceived usefulness for learning. Patterns were examined across the complete corpus and within each learning-modality group; pre- and post-intervention test reflections were also compared. The TAM was not used as an a priori coding framework. Correspondence between the qualitative categories and TAM concepts such as perceived usefulness, ease of use, and attitude was considered subsequently when interpreting the qualitative and quantitative findings together.

#### Affective experience and engagement: “A whole different and fun world”

Participants described differing affective experiences with the four learning resources. Several VR participants emphasised novelty, enjoyment, and visual interaction. One described VR as “a whole different and fun world” that made learning “more easy and fun” (VR10), while another characterised it as “interesting and less boring compared to books” (VR06). These comments suggest that, for some participants, the immersive and interactive format fostered enjoyment and interest. This response was not universal, however, and positive affect did not necessarily indicate that participants found the available content sufficient for the knowledge test.

Positive comments about AMBOSS^®^ more often emphasised efficiency and practical usefulness than novelty. One participant stated that “it feels good to use AMBOSS^®^ unlike books and slides,” while describing it as “easy and very fast to use” (AMB03). Positive engagement with AMBOSS^®^ thus appeared to arise principally from its perceived efficiency and organisation.

Comments from the Book and Internet groups were more mixed. Some participants valued the available information, whereas others emphasised the effort needed to locate and process relevant content. One Book participant referred to the “lot of effort and time” required (BK02), and an Internet participant described searching for information as “time consuming … and a bit stressful” (INT06).

Overall, the comments indicate different forms of positive and negative experience rather than a simple hierarchy of engagement: enjoyment and novelty were particularly evident in some VR responses, efficiency in AMBOSS^®^ responses, and effortful information processing in some Book and Internet responses.

#### Perceived information quality: “The information was too bulky”

Information quality was a prominent aspect of participants’ experiences. Comments concerned both the presentation of information, particularly its organisation and conciseness, and its perceived relevance and completeness for answering the knowledge-test questions.

AMBOSS^®^ participants frequently praised the organisation and accessibility of its content. One participant valued that “the information is well organized for easier access and well explained for easier understanding” (AMB01). Another emphasised the usefulness of integrated explanations, noting that “most of the difficult terms are also explained on the very app” and that there was “no need to Google” unfamiliar terminology (AMB07). These comments suggest that the curated structure of AMBOSS^®^ reduced the need to search across multiple sources.

Some Book and Internet participants described a different difficulty: the resources contained substantial information, but identifying and synthesising the most relevant material required additional effort. One Book participant described the information as “too bulky” and “not as summarised as one can use” (BK02). Similarly, an Internet participant found it “quite difficult to navigate and search for the right and correct information because you have an array of options” (INT10). These comments indicate that the amount and organisation of information, rather than its complete absence, could make the resources more difficult to use within the available study period.

In the VR group, concerns focused more directly on perceived content completeness and alignment with the assessment. One participant reported that the resource “lacked some of the needed structures like the lymph” (VR08), while another stated that “some” of the questions “were not found in the VR” (VR03). The latter participant also acknowledged that additional time using the resource might have allowed more relevant information to be discovered, an observation consistent with the proctor’s report that most VR participants ended their sessions well before the 30-minute maximum. Perceived content gaps may therefore have reflected actual differences in coverage, difficulty locating information within the application, limited familiarity with navigation, insufficient exploration time, or a combination of these factors.

Overall, the comments suggest different information-quality challenges across modalities: AMBOSS^®^ was valued for organised and readily accessible explanations; the Book and Internet resources were sometimes experienced as extensive but insufficiently curated; and VR users raised concerns about the visibility or perceived completeness of content relevant to the assessment. These learner perceptions should not be interpreted as objective evidence that specific content was absent, because the pre-study review addressed content presence in the fixed-content resources but not how readily novice users could locate and interpret it within the study period.

#### System quality and ease of use: “I found the VR hard to navigate”

Ease of use formed an important component of participants’ experiences, but the nature of usability differed between learning resources. AMBOSS^®^ participants frequently described the platform as straightforward and efficient. One participant characterised it as “easy and very fast to use” and valued the availability of “diagrams and illustrations” that made it easier to use (AMB03). Other comments similarly associated its organisation and navigability with rapid access to relevant content.

Comments about VR usability were more mixed. Several participants described the application positively, but some reported difficulty navigating an unfamiliar interface. One participant stated that the VR application was “hard to navigate through” (VR13). Another believed that “more time” was needed because the “new way of learning needed much time to adapt and get used to it” (VR07). These comments suggest that some participants required additional orientation or familiarisation before they could use the application confidently. Because the learning sessions were self-paced, actual time-on-task was not recorded, and 30 min represented a maximum rather than a fixed exposure period, the extent to which navigation difficulties resulted from limited time cannot be determined.

Internet-resource users described both resource-navigation and contextual technical barriers. Some found it difficult to identify appropriate information among numerous available websites. One participant also reported that “the network … wasn’t good” and stated, “I don’t know much about the computer” (INT17), indicating that connectivity and prior digital familiarity could affect the learning experience. Another participant observed that the resource was “impossible to use when there is no internet connection” (INT12). Although such infrastructure-related comments were relatively infrequent, they illustrate practical dependencies that did not apply to the offline VR and Book resources.

Overall, the comments indicate modality-specific usability considerations: AMBOSS^®^ was generally experienced as organised and readily navigable; some VR participants required greater familiarisation with the interface; and Internet use depended on participants’ ability to navigate multiple sources as well as on connectivity and digital familiarity. Book-related usability concerns primarily involved the effort required to locate and synthesise relevant information and are therefore considered under information quality.

#### Perceived test preparedness and assessment alignment: “Interesting … but I hardly got to see these organs”

Participants’ comments suggest that perceived preparedness for the post-test was related to whether they could readily locate information that appeared relevant to the assessment. These comments represent participants’ perceptions rather than a validated measure of confidence or objective evidence of assessment alignment.

Several AMBOSS^®^ comments linked perceived preparedness to the organisation and apparent completeness of the platform. One participant stated that “AMBOSS^®^ has the necessary data needed to pass this test” (AMB06). Another described it as “a very organised platform” that provided “information needed to answer any question” (AMB12). Other AMBOSS^®^ comments similarly emphasised the availability of the main anatomical information needed for test preparation, suggesting that some participants perceived a relatively direct connection between the platform’s content and the assessment.

VR comments illustrated that enjoyment and perceived preparedness did not necessarily coincide. One participant reported that the resource helped with preparation because “I could see the parts of anatomy” (VR02). Another described VR as engaging but added that, regarding terms appearing in the test, “I hardly got to see these organs” (VR06). Together, these comments show that visual engagement could support learning while still leaving some participants uncertain about whether they had located all assessment-relevant content.

Book participants reported mixed experiences. Some described the textbook as useful and considered the post-test fair, whereas others found it difficult to locate relevant information within the available time. One participant stated that it was “not easy to access the information required” (BK07), and another reported that “time wasn’t enough” because the relevant page was not found early in the session (BK11). These comments point to information-access and time-management challenges rather than demonstrating a general mismatch between the textbook and the assessment.

Internet participants likewise described varied experiences. Some reported that Internet searching helped them prepare and improved their understanding, while others found it difficult to identify the most appropriate information among numerous sources. One participant described difficulty searching “for the right and correct information because you have an array of options” (INT10), and another reported being unable to study because of a “poor internet connection” (INT26). Perceived preparedness with the Internet resource therefore appeared to depend partly on search efficiency and connectivity.

Overall, the comments suggest that participants felt better prepared when relevant information appeared complete, clearly organised, and readily accessible. Conversely, difficulty locating content, navigating the resource, or accessing the Internet could reduce perceived preparedness. The findings do not establish a hierarchy of learner confidence across modalities, but they illustrate how enjoyment, usability, information accessibility, and perceived assessment alignment could interact in individual learning experiences.

## Discussion

### Principal finding

This exploratory randomised study provides preliminary, contextually grounded evidence concerning immediate knowledge outcomes and learner experiences with four anatomy-learning resources in a resource-constrained setting. The primary baseline-adjusted analysis did not detect a statistically significant overall difference in post-test knowledge scores between VR, AMBOSS^®^, textbook materials, and self-directed internet search.

Quantitative differences in acceptance were more limited than initially apparent. Although unadjusted comparisons suggested differences in five of the six assessed domains, only Attitude remained statistically significant after adjustment for multiple testing, and that result was not robust in a sensitivity analysis excluding one participant with a provisional group assignment. The subsequent pairwise comparison indicated more favourable Attitude ratings for AMBOSS than for the self-directed internet search. The remaining quantitative findings did not establish significant differences in perceived usefulness, ease of use, behavioural intention, enjoyment, or job relevance.

The written comments nevertheless indicated that participants experienced the resources differently. Some VR participants emphasised novelty, visualisation, and enjoyment, while others reported navigation difficulties and uncertainty about locating assessment-relevant content. AMBOSS^®^ participants frequently valued the organisation and accessibility of its information. Some Book and Internet participants described locating and synthesising relevant material as effortful, although responses within these groups were mixed and included positive experiences.

The principal contribution is therefore not evidence that the modalities produced equivalent learning or that one technology was generally preferred. Rather, the findings suggest that information organisation, content accessibility, interface familiarity, and infrastructure may shape learner experience independently of immediate knowledge-test performance. These observations provide hypotheses and implementation considerations for a larger, adequately powered comparative study using explicitly aligned content, recorded exposure time, and validated outcome instruments.

### Interpreting the quantitative and qualitative findings

The absence of a statistically significant overall difference in baseline-adjusted post-test scores should not be interpreted as evidence that the four modalities were equivalent or equally effective. The study was exploratory, had modest group sizes, and did not include a no-intervention control. It therefore provides uncertain comparative estimates of immediate knowledge outcomes rather than definitive evidence about the effectiveness of each resource.

The qualitative findings add context to these estimates by illustrating aspects of the learning experience that participants noticed and valued. They cannot establish the mechanisms responsible for the observed knowledge scores, because the data consisted of brief written comments without follow-up questioning, and relevant factors such as time-on-task, prior familiarity, and cognitive load were not measured. Nevertheless, the comments indicate that participants evaluated the resources using criteria extending beyond immediate test performance.

Some VR participants emphasised novelty, visualisation, interactivity, and enjoyment, while others described navigation difficulties and uncertainty about locating assessment-relevant content. AMBOSS^®^ participants frequently valued organised information, integrated explanations, and efficient access to relevant material. Book and Internet experiences were more mixed than a simple positive–negative distinction suggests: some participants considered these resources useful, whereas others described the effort involved in locating, filtering, and synthesising relevant information.

These observations suggest possible differences in how learners experienced the four resources, even though the study did not provide conclusive evidence of between-group differences in immediate knowledge outcomes. The qualitative material should therefore be understood as identifying potentially important dimensions of implementation and learner experience, not as explaining why the modalities produced particular knowledge scores. Future studies should assess these proposed relationships directly using aligned content, recorded exposure time, validated engagement and cognitive-load measures, and adequately powered learning outcomes.

### Perceived usefulness and enjoyment as interpretive dimensions

The TAM provides useful terminology for interpreting participants’ evaluations of the learning resources [15–17]. However, the present study did not test the causal relationships specified by TAM or identify determinants of actual technology adoption. The adapted questionnaire was administered after a single learning session, had not been validated in this population, and included two domains with low internal consistency. The findings should therefore be interpreted as immediate acceptance ratings rather than evidence of enduring adoption.

The qualitative comments nevertheless provide conceptually relevant context. AMBOSS^®^ participants frequently valued organised information, integrated explanations, and efficient access to relevant content, which overlap conceptually with perceived usefulness and ease of use. Some VR participants emphasised novelty, visualisation, and enjoyment, although others reported navigation difficulties and uncertainty about locating assessment-relevant information. Book and Internet responses were mixed rather than uniformly negative.

### Perceived preparedness rather than an “Illusion of Competence”

The study cannot determine whether perceived and actual knowledge differed because it did not use a validated confidence measure or compare participants’ self-assessments with their individual test scores. Self-assessed knowledge improvement was also similar across groups, with a median of 2.0 in every group, although the IQRs differed (VR: 2.0–3.0; AMBOSS^®^: 1.0–3.0; Book: 1.0-2.25; Internet: 1.0–2.0; lower scores indicate stronger agreement).

Some written comments illustrated that enjoyment and perceived preparedness did not always coincide. One VR participant described the experience as interesting but reported difficulty locating anatomical structures referenced in the test. Such comments suggest uncertainty about content accessibility or assessment alignment; they do not establish inflated confidence or inaccurate self-assessment.

Other publications have demonstrated that self-assessment and objective performance can diverge in health-professions education [21], and immersive-learning research has shown that greater presence or enjoyment does not necessarily produce greater learning [22, 23]. These findings support examining the alignment between perceived and actual knowledge in future VR studies. Future studies could assess this directly by collecting prospective confidence ratings for individual test items and comparing them with objective performance.

### Usability and contextual requirements

Participants’ comments identified several resource-specific usability considerations. AMBOSS was frequently described as organised and readily navigable. Some VR participants reported that they required additional time to become familiar with the interface or had difficulty locating relevant content. Internet-resource users described challenges associated with navigating multiple sources, connectivity, and limited computer familiarity. Book-related challenges primarily concerned the time and effort required to locate and synthesise relevant information.

These observations do not establish that VR usability depended on hard- or software reliability, because participants did not report systematic headset or software failures. Likewise, the study did not assess electricity supply, maintenance costs, institutional technical capacity, or cost-effectiveness. The principal VR-related concerns documented here involved interface familiarisation, perceived content accessibility, and limited or unrecorded exploration time. Broader reviews have identified equipment costs, accessibility, technical support, user training, and availability of contextually appropriate content as potential barriers to immersive-technology implementation in resource-constrained settings [5, 7, 9], alongside wider constraints on digital participation such as connectivity and digital literacy [24]. The pre-study review indicated that the assessed concepts could be located within each of the three fixed-content resources, although no structured audit record is available. Participants’ reports of VR content gaps therefore cannot establish that content was objectively absent. They may instead reflect differences in content visibility, terminology, navigation, depth of presentation, time spent using the application, or participants’ ability to identify relevant material. This distinction reinforces the importance of evaluating not only whether educational content is technically present, but also whether novice learners can readily locate and interpret it.

The positive affective comments made by some VR participants are consistent with research showing that immersive environments can generate presence, interest, and enjoyment [22, 23]. Nevertheless, the same experimental work has shown that increased immersion or presence does not necessarily improve learning. These findings provide a relevant interpretive context but do not establish that the same cognitive or affective mechanisms operated in this sample.

Reviews of immersive technologies in low- and middle-income countries have highlighted accessibility, device limitations, technical capacity, and the availability of appropriate content as implementation concerns [5, 7, 9]. The present study adds preliminary learner-level observations from a Zambian medical-education setting, particularly regarding interface familiarisation, information accessibility, and perceived assessment alignment. It does not demonstrate the prevalence of wider infrastructural barriers or establish a new implementation model.

### Practical implications

The findings are not sufficiently definitive to recommend investment in one modality over another or to support claims of proven effectiveness, return on investment, or scalable adoption. They nevertheless suggest several practical considerations.

First, learners appeared to value resources that made relevant information easy to locate, interpret, and connect with the assessment objectives. Curated textbook extracts and web resources could potentially be improved through clearer signposting, removal of unnecessary material, direct links, and explicit mapping to learning objectives, rather than being dismissed as inherently inferior.

Second, AMBOSS^®^ was often valued for its organisation, but its use here required an institutional licence and reliable internet connectivity. Participants accessed linked library articles and anatomical illustrations; they did not use embedded question banks or immediate performance feedback, which have been examined separately in relation to examination performance [25]. Claims concerning retrieval practice, calibrated self-assessment, minimal infrastructure requirements, or cost-effectiveness are therefore not supported by this intervention.

Third, future evaluation of VR should include a standardised orientation, explicit mapping between application content and curricular objectives, and systematic recording of time-on-task. Researchers should distinguish between content being absent and learners being unable to locate it. Implementation planning should also consider headset capacity, hygiene, technical support, software maintenance, and supervision staffing, although the costs and feasibility of these were not evaluated here.

Finally, any subsequent comparative trial should use explicitly aligned content, record exposure and navigation time, include validated measures of usability, engagement, cognitive load, and confidence, and assess longer-term retention.

### Limitations

The study was exploratory, with no a priori sample-size calculation; 59 participants across four groups (12–17 per group) gave limited power and wide confidence intervals, so non-significant results are not evidence of equivalence. There was no no-intervention control, so pre–post gains may partly reflect repeated exposure to the same test rather than the assigned resource. Randomisation was not concealed, and participants and proctors could not be blinded to visibly different resources, although the knowledge-test scorer was blinded.

Content comparability could not be fully established: the pre-study review indicated that assessed concepts could be located in each of the three fixed-content resources, but it was not documented as a structured mapping, did not establish that depth, terminology, organisation, or ease of access were equivalent for novice learners, and could not be applied to the internet group. Sessions were self-paced to a 30-minute maximum and time-on-task was not recorded, so differences in exposure, search requirements, and interface familiarity may have affected outcomes, particularly as some VR participants ended well before the maximum while others reported needing more time.

Both instruments were unvalidated. The 10-item knowledge test was not piloted and showed low internal consistency (KR-20 = 0.32 pre-test, 0.54 post-test); identical items were used at both time points, permitting recall effects; and the post-test assessed only short-term factual knowledge, not retention, spatial understanding, or transfer. The acceptance questionnaire retained the title “Virtual Reality Acceptance Questionnaire” for all groups, which may have framed non-VR participants’ responses; two near-duplicate items were identified and excluded only during revision; Perceived Ease of Use and Perceived Enjoyment showed low internal consistency; and Job Relevance was a single item.

The qualitative component comprised brief written responses without opportunity for follow-up, analysed by one researcher without double-coding, disagreement resolution, participant validation, or inter-rater reliability assessment; no claim of saturation is made. Random group assignments were generated by the study lead in Microsoft Excel and were not concealed from the study lead. The original allocation spreadsheet was not retained, preventing independent verification of the exact allocation procedure during manuscript revision. Participants and proctors could not be blinded after the visibly different resources were disclosed, although the knowledge-test scorer was blinded to group assignment.

Finally, costs, maintenance, faculty workload, and institutional readiness were not evaluated, so the study cannot establish feasibility or scalability beyond this setting. Content comparability could not be assessed for the Internet group, in which participants selected their own online sources; the informal pre-study content review therefore covered the VR, AMBOSS^®^, and textbook resources only.

### Future research

Future studies should use an adequately powered, prospectively registered design with a prespecified primary outcome, formal allocation concealment, and a statistical analysis plan addressing multiple comparisons. Fewer intervention groups, or a sample size powered for clearly defined primary contrasts, may be preferable. Including a usual-teaching or no-additional-intervention comparison would help distinguish intervention-related learning from testing and study-participation effects.

Comparative studies should use explicitly mapped content and distinguish between content being technically present and being readily discoverable by novice learners. Exposure time should be standardised or recorded systematically, and digital platforms should capture navigation patterns, content accessed, and time spent on individual activities. For VR, studies should evaluate the amount and form of orientation needed before learners can use the interface effectively.

Future assessment should employ a longer, piloted knowledge instrument with established content validity, item performance, and reliability. Outcomes should include delayed retention and, where relevant, spatial understanding, application, transfer, or practical skills; immersive technologies have also been examined as assessment tools, which raises separate measurement considerations [27]. Validated measures of usability, engagement, presence, cognitive load, and technology acceptance would permit more reliable examination of how learner experience relates to performance.

A larger qualitative component involving interviews or focus groups could explore how learners navigate different resources, interpret content alignment, and experience infrastructural or organisational constraints. Multiple analysts, a documented coding framework, and explicit reflexive procedures would strengthen this work.

Multi-institutional studies should also examine implementation requirements, including licensing, headset capacity, connectivity, maintenance, infection-control procedures, technical support, staff time, and cost-effectiveness; implementation-science frameworks offer established approaches for evaluating adoption and scale-up [26]. Curriculum-aligned VR content could be developed through user-centred co-design with local students and educators. Hybrid approaches combining visualisation with structured knowledge retrieval or feedback may be worth comparing, but their benefits should be evaluated empirically rather than assumed.

## Conclusion

In this small exploratory randomised study, the primary baseline-adjusted analysis did not detect a statistically significant overall difference in short-term anatomy knowledge scores between VR, AMBOSS^®^, textbook materials, and self-directed internet search. Given the modest group sizes and wide confidence intervals, this result does not establish equivalent effectiveness or exclude potentially meaningful differences between modalities.

After adjustment for multiple comparisons, quantitative acceptance differences were limited to more favourable Attitude ratings for AMBOSS^®^ than for internet search. Written comments provided additional context: some VR participants valued visualisation, novelty, and enjoyment but reported navigation and content-access concerns; AMBOSS^®^ participants frequently valued organised and accessible information; and textbook and internet experiences were mixed, with some participants describing difficulty locating and synthesising relevant material.

The findings should therefore be interpreted as preliminary evidence concerning learner experience and implementation considerations rather than proof of comparative effectiveness, technology adoption, or scalability. They highlight content accessibility, curricular alignment, interface familiarisation, exposure time, and infrastructure as priorities for future evaluation. Larger, prospectively designed studies using validated instruments and longer-term outcomes are needed before recommendations can be made regarding the role of VR or other digital resources in anatomy education in Zambia or comparable settings.

## Supplementary Information

Below is the link to the electronic supplementary material.

Supplementary Material 1

## Data Availability

The datasets generated and analysed during the current study are available from the corresponding author on reasonable request. The analysis code and blank templates for data reconciliation are publicly available at https://doi.org/10.5281/zenodo.21895022. Participant-level data, completed correction tables and the identifier crosswalk are not included in the public repository. The original study files contain student identifiers and are held under restricted access; access is subject to the conditions of the ethics approvals.
