# Supplementary material for: Beyond knowledge gains - learner experience and technology acceptance of virtual reality for anatomy education in Zambia: a randomized exploratory study
Source: Adv Simul (Lond). 2026 Oct 31;11:81. doi: 10.1186/s41077-026-00493-0 (PMC13723937; doi:10.1186/s41077-026-00493-0)
Supplement: Supplementary file 1 [file 41077_2026_493_MOESM1_ESM.docx]

**Appendix A -** 10-item pre/post test questions (with correct answers)

**Appendix B –** Questionnaire based on the technology acceptance model (TAM), adapted for Virtual Reality and digital learning resources.

**Appendix C.** CONSORT 2025 checklist

| **Section / topic** | **No** | **CONSORT 2025 checklist item** | **Reported in** |
| --- | --- | --- | --- |
| *Title and abstract* | 1a | Identification as a randomised trial | Title |
|  | 1b | Structured summary of the trial design, methods, results, and conclusions | Abstract |
| *Open science — trial registration* | 2 | Name of trial registry, identifying number (with URL) and date of registration | Methods, Study design — not registered; reason given |
| *Protocol and statistical analysis plan* | 3 | Where the trial protocol and statistical analysis plan can be accessed | Methods, Study design — no protocol or SAP; acknowledged as a limitation |
| *Data sharing* | 4 | Where and how the individual de-identified participant data, statistical code and any other materials can be accessed | Availability of data and materials |
| *Funding and conflicts of interest* | 5a | Sources of funding and other support, and role of funders | Funding |
|  | 5b | Financial and other conflicts of interest of the manuscript authors | Competing interests |
| *Introduction — background* | 6 | Scientific background and rationale | Introduction |
| *Objectives* | 7 | Specific objectives related to benefits and harms | Introduction, final paragraph |
| *Methods — patient and public involvement* | 8 | Details of patient or public involvement in the design, conduct and reporting | Not applicable — educational study without patient involvement |
| *Trial design* | 9 | Description of trial design including type of trial, allocation ratio, and framework | Methods, Study design and Randomisation — four-arm parallel design; equal-probability allocation (intended 1:1:1:1), without fixed group sizes |
| *Changes to trial protocol* | 10 | Important changes after the trial commenced, including analyses that were not prespecified, with reason | Methods, Study design and Data analysis — analyses specified during revision |
| *Trial setting* | 11 | Settings and locations where the trial was conducted | Methods, Participants and study setting |
| *Eligibility criteria* | 12a | Eligibility criteria for participants | Methods, Participants and study setting |
|  | 12b | If applicable, eligibility criteria for sites and for individuals delivering the interventions | Methods, Interventions — proctor roles |
| *Intervention and comparator* | 13 | Intervention and comparator with sufficient details to allow replication | Methods, Interventions and Pre-study content review — Internet search was self-directed; no sites were prescribed, accessed content was not recorded, and no structured content mapping or audit record was created or retained |
| *Outcomes* | 14 | Prespecified primary and secondary outcomes, including measurement variable, analysis metric, aggregation, and time point | Methods, Data collection instruments and Data analysis |
| *Harms* | 15 | How harms were defined and assessed | Not applicable — no harms anticipated or assessed in an educational study |
| *Sample size* | 16a | How sample size was determined, including all assumptions | Methods, Data analysis — no a priori calculation; acknowledged |
|  | 16b | Explanation of any interim analyses and stopping guidelines | Not applicable — none conducted |
| *Randomisation: sequence generation* | 17a | Who generated the random allocation sequence and the method used | Methods, Randomisation — study lead (SB); Excel-generated random group number 1–4 for each participant |
|  | 17b | Type of randomisation and details of any restriction | Methods, Randomisation — participant list organised by gender and sorted by age before unrestricted random group-number generation; no formal stratification, blocking or sequential allocation |
| *Allocation concealment mechanism* | 18 | Mechanism used to implement the allocation sequence, describing steps to conceal it | Methods, Randomisation — no formal concealment; SB knew the assignments; no sealed envelopes |
| *Implementation* | 19 | Whether personnel who enrolled and assigned participants had access to the sequence | Methods, Randomisation and Study procedure — SB generated and accessed the assignments; the student secretary and class representative received attendance times only |
| *Blinding* | 20a | Who was blinded after assignment to interventions | Methods, Randomisation — participants and proctors were informed after the pre-test; the knowledge-test scorer was blinded |
|  | 20b | If blinded, how blinding was achieved and similarity of interventions | Methods, Randomisation — coded tests enabled assessor blinding; visibly different interventions prevented participant and proctor blinding |
| *Statistical methods* | 21a | Statistical methods used to compare groups for primary and secondary outcomes | Methods, Data analysis |
|  | 21b | Definition of who is included in each analysis, and in which group | Methods, Data analysis; Results, Descriptive study results |
|  | 21c | How missing data were handled in the analysis | Methods, Data analysis — no imputation |
|  | 21d | Methods for any additional analyses, distinguishing prespecified from post hoc | Methods, Data analysis — all post hoc |
| *Results — participant flow* | 22a | For each group, numbers randomly assigned, receiving intended intervention, and analysed | Figure 1; Results |
|  | 22b | For each group, losses and exclusions after randomisation, with reasons | Figure 1; Limitations |
| *Recruitment* | 23a | Dates defining the periods of recruitment and follow-up | Methods, Participants and study setting — July 2022, single day |
|  | 23b | If relevant, why the trial ended or was stopped | Not applicable |
| *Intervention and comparator delivery* | 24a | Intervention and comparator as actually administered, including fidelity | Methods, Interventions and Study procedure — self-directed Internet search; no prescribed sites; attendance times communicated without resource assignments |
|  | 24b | Concomitant care received during the trial for each group | Not applicable |
| *Baseline data* | 25 | Table showing baseline demographic and clinical characteristics for each group | Table 1 |
| *Numbers analysed, outcomes and estimation* | 26 | For each outcome, by group: number analysed, number with available data, result, estimated effect size and precision | Tables 2 and 3; Results |
| *Harms* | 27 | All harms or unintended events in each group | Not applicable — none reported |
| *Ancillary analyses* | 28 | Any other analyses performed, distinguishing prespecified from post hoc | Results; Methods, Data analysis — sensitivity analyses |
| *Discussion — interpretation* | 29 | Interpretation consistent with results, balancing benefits and harms | Discussion, Principal finding |
| *Limitations* | 30 | Trial limitations, addressing bias, imprecision, generalisability and multiplicity | Limitations — no allocation concealment; original allocation spreadsheet not retained; no formal content mapping; one qualitative analyst |

**Appendix D.** COREQ checklist

| **No** | **COREQ item** | **Reported in / status** |
| --- | --- | --- |
|  | **Domain 1: Research team and reflexivity** |  |
| 1 | Interviewer/facilitator: which author conducted the interview or focus group? | Not applicable — written open-text survey responses; the corpus was analysed by JN |
| 2 | Credentials: what were the researcher's credentials? | Methods, Data analysis — health-psychology researcher, Heidelberg Institute of Global Health |
| 3 | Occupation: what was their occupation at the time of the study? | Methods, Data analysis |
| 4 | Gender: was the researcher male or female? | Not reported |
| 5 | Experience and training: what experience or training did the researcher have? | Methods, Data analysis — health-psychology background reported; specific qualitative training not documented |
| 6 | Relationship established prior to study commencement? | No relationship; the analyst was not present during data collection |
| 7 | Participant knowledge of the interviewer | Not applicable — no interviewer contact |
| 8 | Interviewer characteristics reported | Methods, Data analysis |
|  | **Domain 2: Study design** |  |
| 9 | Methodological orientation and theory | Methods, Data analysis — qualitative content analysis, sequential deductive–inductive |
| 10 | Sampling: how were participants selected? | Methods, Participants — all study participants were invited to respond |
| 11 | Method of approach | Methods, Study procedure — written prompts within the post-intervention questionnaire |
| 12 | Sample size: how many participants were in the study? | Results — 211 responses from 64 participants |
| 13 | Non-participation: how many people refused or dropped out? | Results — not all participants answered every prompt; counts reported by prompt |
| 14 | Setting of data collection | Methods, Study procedure — main study room, proctored |
| 15 | Presence of non-participants | Not applicable |
| 16 | Description of sample | Table 1 |
| 17 | Interview guide: were questions or prompts provided? | Four open-text prompts; wording reproduced in Appendix B |
| 18 | Repeat interviews | Not applicable |
| 19 | Audio/visual recording | Not applicable — responses were written |
| 20 | Field notes | Not applicable |
| 21 | Duration of interviews or focus groups | Not applicable |
| 22 | Data saturation: was saturation discussed? | Methods, Data analysis — no claim of saturation is made |
| 23 | Transcripts returned to participants for comment | Not applicable — participants could not be recontacted |
|  | **Domain 3: Analysis and findings** |  |
| 24 | Number of data coders | Methods, Data analysis — one analyst; no double coding |
| 25 | Description of the coding tree | Results, Qualitative analysis — three principal categories described |
| 26 | Derivation of themes: were themes identified in advance or from the data? | Methods, Data analysis — evaluative direction deductive; reasons coded inductively |
| 27 | Software used to manage the data | Methods, Data analysis — Microsoft Word and XMind |
| 28 | Participant checking: did participants provide feedback on the findings? | Not applicable — no recontact was possible |
| 29 | Quotations presented and identified | Results, Qualitative analysis — quotations with participant identifiers |
| 30 | Data and findings consistent | Results, Qualitative analysis |
| 31 | Clarity of major themes | Results, Qualitative analysis — presented as separate subsections |
| 32 | Clarity of minor themes | Results, Qualitative analysis |
